# Convolutional Neural Network–Machine Learning Model: Hybrid Model for Meningioma Tumour and Healthy Brain Classification

**DOI:** 10.3390/jimaging10090235

**Published:** 2024-09-20

**Authors:** Simona Moldovanu, Gigi Tăbăcaru, Marian Barbu

**Affiliations:** 1 Department of Computer Science and Information Technology, Faculty of Automation, Computers, Electrical Engineering and Electronics, “Dunarea de Jos” University of Galati, 800146 Galati, Romania; 2 The Modelling & Simulation Laboratory, “Dunarea de Jos” University of Galati, 47 Domneasca Str., 800008 Galati, Romania; 3 Department of Automatic Control and Electrical Engineering, Faculty of Automation, Computers, Electrical, Engineering and Electronics, “Dunarea de Jos” University of Galati, 800146 Galati, Romania; gigi.tabacaru@ugal.ro (G.T.); marian.barbu@ugal.ro (M.B.)

**Keywords:** meningioma tumour, convolutional neural networks, machine learning, transfer learning

## Abstract

This paper presents a hybrid study of convolutional neural networks (CNNs), machine learning (ML), and transfer learning (TL) in the context of brain magnetic resonance imaging (MRI). The anatomy of the brain is very complex; inside the skull, a brain tumour can form in any part. With MRI technology, cross-sectional images are generated, and radiologists can detect the abnormalities. When the size of the tumour is very small, it is undetectable to the human visual system, necessitating alternative analysis using AI tools. As is widely known, CNNs explore the structure of an image and provide features on the SoftMax fully connected (SFC) layer, and the classification of the items that belong to the input classes is established. Two comparison studies for the classification of meningioma tumours and healthy brains are presented in this paper: (i) classifying MRI images using an original CNN and two pre-trained CNNs, DenseNet169 and EfficientNetV2B0; (ii) determining which CNN and ML combination yields the most accurate classification when SoftMax is replaced with three ML models; in this context, Random Forest (RF), K-Nearest Neighbors (KNN), and Support Vector Machine (SVM) were proposed. In a binary classification of tumours and healthy brains, the EfficientNetB0-SVM combination shows an accuracy of 99.5% on the test dataset. A generalisation of the results was performed, and overfitting was prevented by using the bagging ensemble method.

## 1. Introduction

According to Cancer Research UK, over 100 different types of brain tumours (BTs), both malignant and benign, can develop in the human brain. The statistics provided by the WHO show that the average survival rate is only 35%, and in 2019, approximately over 700,000 people were diagnosed with a brain tumour [1]. In 2020, over 87,000 patients were diagnosed with brain tumours, and in 2021, over 84,170 cases were reported [2]. A report indicated by the WHO shows that BTs represent less than 2% of cancers in humans [3].

In order to detect the BTs, computed tomography (CT) and MRI (magnetic resonance imaging) techniques are used to scan the brain and display cerebral matter as texture pixels.

Machine and deep learning are two subfields of artificial intelligence. These tools can be used individually or together to classify the different types of diseases. Moreover, the detection process can be integrated into an automatic or semi-automatic model in order to achieve early detection of BTs, where the main advantage is an increase in survival rates [4]. Several efforts have been made to develop highly accurate and robust methods for the classification of BTs from CT images. The methods can be divided into the following categories: various pre-trained CNNs [4,5,6,7,8,9,10,11,12,13,14,15,16] and hybrid methods [8,9,10,17]. 

In our proposed framework, three deep learning and ML tools were used, and an evaluation of classification in the context of accuracy and area under the curve (AUC) was performed. This combination of CNNs and ML tools improves upon the low accuracy obtained for the base model, and it leads to the best classification accuracy when the ML tools are trained on features generated by CNNs.

The main objective of this paper is to compare different CNNs and ML models to identify the best combination for distinguishing between meningioma tumour and healthy brain tissue, using information extracted from brain MRI images. The secondary objective was to optimize a classical CNN in an ablation process and to obtain efficient results when ML models are trained with the features that pertain to an SFC layer.

The following contributions have been made in order to fulfil the paper’s objectives:

- Selection of an adequate brain MRI dataset that contains meningioma tumours and healthy brains;
- Development of an original CNN.
- Selection of two pre-trained models (i.e., EfficientNetV2B0 and DenseNet169) according to the top five accuracies obtained on an ImageNet dataset.
- Three CNN models’ features were used to train RF, KNN, and SVM machine learning classifiers.
- We measured classification accuracy and ROC-AUC concerning the base CNN and transfer learning models.
- A bagging ensemble approach with 5-fold cross-validation was employed to prevent overfitting and aggregate their results.
- In the last part of the study, the detection of the best hybrid model that can differentiate between the studied classes was performed.

The worrying statistics that show the global incidence of brain tumours is the primary motivation. Significant achievements toward advanced and integrated AI in enhancing patient quality of life include the integration of AI tools for the classification process into the medical profession and the corroboration of AI tools for better classification.

The limitations of this paper are the high computational power and dependence on a large set of images; in addition, this type of data occupies significant space. 

The current paper is organized as follows in order to achieve the suggested goal and contributions. Section 2 contains a detailed selection of relevant studies that deal with the classification of different tumour types. The methodology is described in Section 3, where a step-by-step breakdown of this paper is given and a detailed flowchart is provided. The study elements, such as dataset, augmentation, feature extraction and evaluation, CNNs, ML models, and performance metrics, are also detailed. Section 4 is assigned to results and discussions. Section 5 contains a comparison with state-of-the-art models, and finally, the conclusions and some future prospects in this field of study are presented in Section 6.

## 2. Related Work

This section reviews prior studies that were conducted to classify brain tumours utilising pre-training with classic architecture, CNNs, and ML models. Numerous papers have already been used as references for the classification of brain MRI images by CNNs because of their superior accuracy.

The studies were carried out on the public databases Kaggle MRI [4,5,6,7,8,9,10,11,12,13,14], SARTAJ, and Br35H [6]. The MRI images were classified by specialists into healthy brains and different tumour types. This database fed CNNs with normal architectures, pre-trained CNNs, or hybrid models of CNNs ML models.

In our study, the original CNN, pre-trained CNNs, and hybrid models were applied. From the scientific literature, only the papers that dealt with this subject were selected. Furthermore, for a thorough overview, Table 1 specifies the references, datasets (with the number of MRI images), models, performance accuracy, and limitations.

This paper proposes a hybrid model because the many studied references proposed only one research direction, such as original CNN [6,13,18], original CNN and pre-trained CNN [4], pre-trained CNN [5,7,11,14], pre-trained CNN and ML [9,10,19,20], and proposed CNN and ML [8,12].

## 3. Proposed Method

This section provides the first explanation of our proposed method in general terms. In the next subsections, we delve deeper into the details of five key sections.

**Figure 1:**
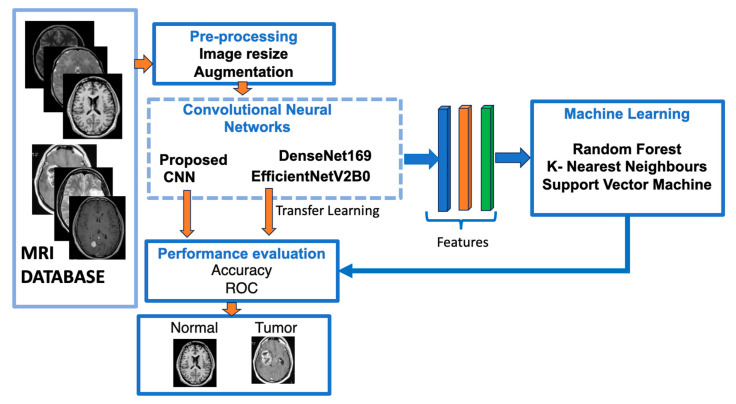
The structure of our suggested models utilising deep learning approaches.

Figure 1 shows a flowchart of our suggested approach for classifying brain meningioma tumours versus normal brains.

**Figure 2:**
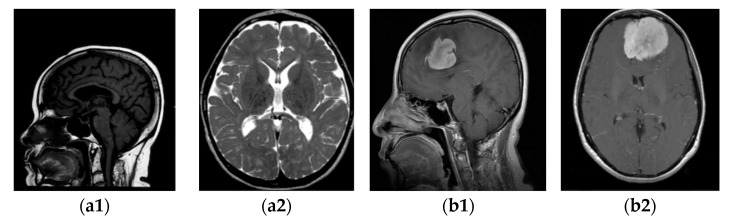
MRI images of two categories: (**a1**,**a2**) without tumour; (**b1**,**b2**) with tumour.

Firstly, we collected the meningioma and normal brain MRI images. The dataset is described in Section 3.1, and some samples are shown in Figure 2.

Before feeding the models, input MRI images underwent pre-processing methods such as MRI resizing and augmentation; an example of image augmentation is shown in Figure 2. Then, as feature extractors, the pre-processed images are fed into two pre-trained CNN models, and a model is built step-by-step. The three machine learning classifiers that were trained using features extracted from previously trained CNN models are discussed in the same section. 

The best mixture was chosen based on the best accuracy, F1-score, the Matthews correlation coefficient and the AUC computed from confusion matrices provided by classifiers. 

### 3.1. Brain MRI Kaggle Dataset

The investigations delineated in this research were executed through the use of a publicly available dataset acquired from the Kaggle platform [21,22]. There were 3264 brain MRI images with different tumour types and without tumours. From this database, the following package was selected in our study: for training images, 822 meningioma tumours and 395 healthy patients, and for testing, 115 meningioma tumours and 105 healthy patients. In the dataset of images with and without tumours, “yes” and “no”, respectively, were labelled. The type of acquisition method is T1-weighted, where contrast images are enhanced by the type of MRI and fat tissue is highlighted in all three planes: axial, sagittal, and coronal. Some samples with (b) and without meningioma tumour (a) in the sagittal (a1, b1) and horizontal (a2, b2) planes are displayed in Figure 2. 

The experiments were performed on a PC with the following architecture: MacBook Pro, Chip Apple, US, California (M1 Pro), memory (16 GB), total number of cores: 10 (8 performance and 2 efficiency), and sourced from Romania.

As a software environment, Google Colab was chosen because it is a powerful tool for Python 3.10 development. The image database in Google Drive was stored, and a connection with Google Colab was performed. The main libraries, i.e., NumPy 1.26.14, Pandas 2.2.1, Tensorflow 2.17.0, Seaborn 0.13.2 and Sklearn 1.4.1 versions, were imported for the showing, processing, and classification of the features and images. 

### 3.2. Data Augmentation and Pre-Processing

**Figure 3:**
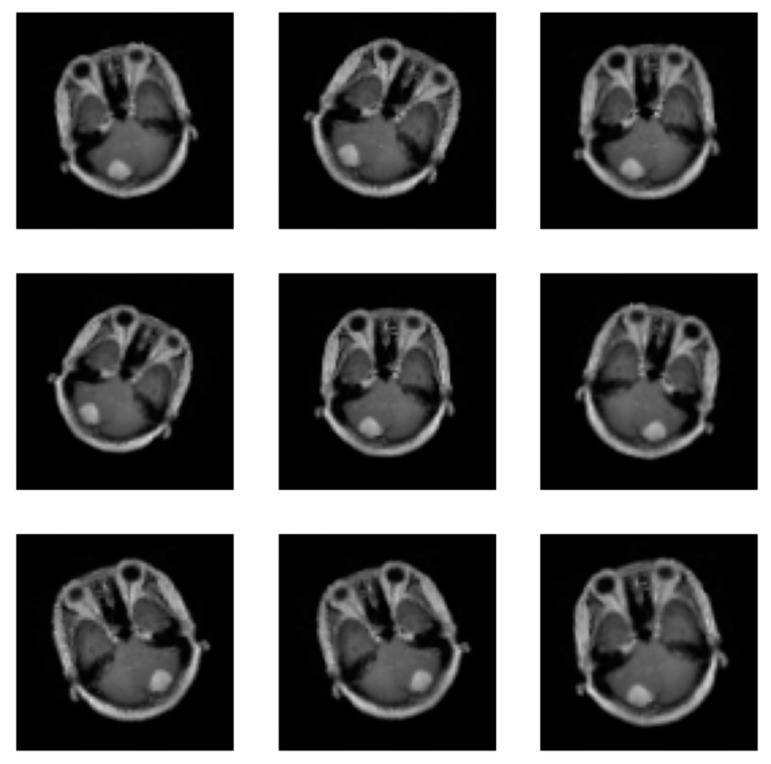
Images augmentation with flip, rotation, and zoom methods.

At this level, deep learning needs a lot of data to learn from. Therefore, data augmentation is employed to increase the quantity of data accessible by altering the original image. Supplementary data can be used to increase the efficacy of the categorized results. Images can undergo the following operations: rotation, scaling, translation, and filtering [23]. This paper uses the random horizontal flip process (the argument represents the probability of the image being flipped at a random angle), random rotation (the argument is a range of degrees by which the image is rotated) and zoom (the argument is a range that configures the percentage of zooming), as shown in Figure 3. All augmentation processes in the training stage are used for the proposed CNN, pre-trained EfficientNetV2B0 and DenseNet169 CNNs, so all CNNs were trained with 2466 meningioma tumour images and 1185 healthy patient images. 

All images were resized for the proposed CNN with a resolution of 80 × 80 and EfficientNetV2B0 and DenseNet169 with a resolution of 224 × 224. 

### 3.3. State-of-the-Art CNN and ML Models

To improve classification, the output of the fully connected layer of the CNN will be processed as an input for ML models. To facilitate feature extraction and improve classification accuracy, a combination of CNNs and ML models was created, and its empirical implementation was monitored. MLs are faster and more efficient than other algorithms because of the hyperparameters and methods integrated that allow them to classify a large number of features; thus, ML models in combination with CNNs are enabled to obtain the best outcomes for brain tumours. In this study, three CNN models were used, and the number of total, trainable, and non-trainable parameters are shown in Table 2. 

Table 3 presents the proposed CNN model, which includes several layers and parameters, and the best architecture was chosen through an ablation process. The selection was based on obtaining low parameters and high accuracy values. 

The pre-trained CNNs were chosen in accordance with Keras applications https://keras.io/api/applications/ (accessed on 10 June 2024). For DenseNet169, the top five accuracies were 93.2% (14.3 million parameters) and 94.3% (7.2 million parameters) for EfficientNetV2B0, respectively.

The empirical optimum architecture of the proposed CNN is highlighted in bold, as shown in Table 3. The number of batches was 20, the image size was 80 × 80, and there were 15 epochs and 3 convolutional layers. A total of 15 layers—one input layer, three max-pooling layers, three dropout layers, one flattening layer, two dense layers, and one output layer—define the depth of the proposed CNN. The original architecture and the parameters for each layer are shown in Table 4.

The roles of each layer and function contained in the proposed CNN are described as follows. The rectified linear unit activation function, called ReLU, is applied in the first dense layer, which captures more complex relationships between input and output layers, performing a threshold operation on each element of the input. In the second dense layer, the SoftMax function is used for a binary classification task [24]. In addition to the CNN model displayed in Table 4, it must be mentioned that each convolution layer contains the ReLU function in order to detect the features within the image. The model also has a pooling layer between two consecutive convolutional layers to reduce the spatial dimensions of the feature maps and a dropout layer for preventing overfitting. In the last two layers, the SoftMax function plays the role of classification, and this function is attached to the dense layer. Thus, an adequate feature extractor is set up for image classification and for reshaping the output into a one-dimensional vector by means of a flattening layer. 

EfficientNetV2B0 pertains to the EfficientNet CNN model category proposed by Tan et al. [25]. The authors came up with the idea of uniformity scales in all dimensions of depth, width, and resolution using a compound coefficient. The next alternative version, EfficientNet-B0, inverts bottleneck residual blocks of MobileNetV2. The architecture of EfficientNet consists of mobile-inverted bottleneck layers, which are a combination of depth-wise separable convolutions and inverted residual blocks. In addition, the model uses squeeze-and-excitation optimisation to further enhance the performance model. This type of CNN is pre-trained on CIFAR-100 and ImageNet.

DenseNet169 (Densely Connected Convolutional Networks) pertains to the DenseNet group proposed by Huang et al. [26] and is pre-trained on the CIFAR-10, CIFAR-100, SVHN, and ImageNet datasets. The main difference between versions is the size and accuracy of the model. The DenseNet169 architecture has the following convolutional, max pooling, dense, and transition layers connected in a feed-forward fashion. Moreover, like the proposed CNN, the architecture of DenseNet169 uses two main activation functions: ReLU and SoftMax.

### 3.4. Training of CNNs and ML Models

After an ablation process for the proposed CNN, the following values for hyperparameters were established: batches of 20, image sizes of 80 × 80, and epochs of 15. The same hyperparameters were kept for EfficientNetV2B0 and DenseNet169. For this CNN, no other resolution size was allowed—with the exception of DenseNet169, where the resolution was 224 × 224. Also, the learning rate for all CNNs was set to 0.001.

For parameter selection for the ML models, a tuning process for the regularisation of hyperparameters was performed; these were selected as a combination of hyperparameter values with the highest accuracy. The values of hyperparameters that meet this criterion for each ML classifier are shown in Table 5. The tuning was performed in accordance with the type and number of hyperparameters for each ML model. Two key hyperparameters influence the performance of RF: the number of trees (NT) and the depth of those trees (MD). We set the depth of trees values to 10, 15, and 20, the number of trees to 50, 100, and 150, and the Gini index as a cost function instead of entropy and log loss. The SVM classifier has two main hyperparameters: C and Gamma. Their values were set to [0.00001, 0.0001, 0.001, 0.01, 0.1, 1, 10, 100, 1000, 10,000]. The SVM algorithm contains linear, sigmoid, and radial basis function kernels. The number of neighbours of the KNN classifier was set from 1 to 10, and alternatively, to find the nearest neighbours, the KDTree or BallTree classes can be used.

### 3.5. Deep Feature Extraction and Classification

Some of the key elements that can influence the outcome are feature extraction, the choice of an adequate classifier, and their classification. In order to extract the features from the images, the researchers employed various ML algorithms to extract the fractal dimension features from breast images (sourced from the U.S.) and classify them with RF, Extra Trees (ET), and XGBoost classifiers [27]. The combination of geometric features and AdaBoost, KNN, DT, and Gaussian Naïve Bayes classifiers produced interesting results when skin lesion images were manipulated [28]. In [29], the PyCaret AutoML performs complex tasks for feature extraction from fundus eye image classification. 

All enumerated studies show that all features were extracted with processed methods from the region of interest or the whole images. This study is an extension of the classification of features produced by CNNs.

The CNN produces features automatically, and the classifiers are applied to the penultimate layer of each CNN. To train each CNN, validation and test sets are selected. The original brain MRI images are fed to the CNNs, and the output is the extracted features from the fully connected layer. Subsequently, healthy and tumour brain susceptibility predictions are performed using SVM, RF, and KNN classifiers with the new training, validation, and test data.

An SVM is an ML tool often used for the classification of features extracted from brain MRI [29]. This tool uses the maximum separating hyperplane [20] to distinguish between the healthy and tumour brains. RF is an ensemble learning method for classification based on decision trees, and together with pre-trained CNNs, it provides a comprehensive performance analysis of the classification problem [27,29,30]. The combination of CNN and KNN effectively detects various forms of brain tumours with promising accuracy [31]. KNN is a non-parametric supervised learning method that has been successfully used to solve classification problems [32].

### 3.6. Feature Evaluation

The performance metrics that we take into consideration in this work are accuracy (ACC) and AUC. The accuracy score is determined using true positives (TP), true negatives (TN), false positives (FP), and false negatives (FN) extracted from the confusion matrix. The ROC metric is obtained by plotting “sensitivity” on the *y*-axis versus “1-specificity” on the *x*-axis. 

The F1-score is an independent metric computed as a harmonic mean between precision and recall. The closer the F1-score value is to one, the more reliable the proposed model is.

The selected dataset is unbalanced, and in this case, the Matthews correlation coefficient (MCC) is proposed because it offers an effective solution to overcoming the class imbalance [28].

The proposed metrics are expressed by Equations (1)–(5):

(1) $$ACC=\frac{TP+TN}{TP+TN+FN+FP}$$

(2) $$precision=\frac{TP}{TP+FP}$$

(3) $$recall=\frac{TP}{TP+FN}$$

(4) $$F1-score=\frac{precision\cdot recall}{precision+recall}$$

(5) $$MCC=\frac{TP\times TN-FP\times FN}{\sqrt{\left(TP+FP\right)\left(TP+FN\right)\left(TN+FP\right)\left(TN+FN\right)}}$$

The ROC curve is used to validate the objective of this paper, i.e., classifying meningioma tumours and healthy brains, as the ROC matrix is independent of the decision threshold and invariant to a priori class probability distributions [33].

## 4. Results and Discussions

In this section, we discuss the success of the three hybrid approaches: proposed CNN-ML model, pre-trained EfficientNetV2B0-ML model, and DenseNet169-ML model.

In the first combination, an original CNN with fifteen layers is proposed, which was trained with the images that pertain to the brain MRI described in Section 3.1. Also, the transfer learning of pre-trained CNNs was performed with features from the same database.

A connection between the ML classifier and the penultimate layer of each CNN was made because the features that pertain to this layer fed the SVM, RF, and KNN classifiers.

The scope of our experiments is to find a hybrid combination to determine the most accurate approach for meningioma tumour brain diagnosis.

In the first experiment, we tested our own CNN in the ablation process. The best combination is highlighted in Table 3, and its architecture is highlighted in Table 4. With the obtained structure, the best accuracy on the test dataset was 0.945.

The second experiment involved the transfer learning of two pre-trained CNNs and verifying their efficiency in terms of accuracy. The initial accuracy on the test dataset without ML tools was 0.985 for EfficientNetV2B0 and 0.981 for DenseNet169, respectively. Next, we found that the accuracy could be improved if the ML classifiers were applied to the features stored on the penultimate layer of each CNN. Hence, the following empirical step was performed. 

The third experiment started with the combinations of CNN and ML models, pre-trained CNN EfficientNetV2B0 and ML models, and DenseNet169 and ML models to verify if the accuracy was improved.

Table 6 below shows the train and validation sets, the combinations of CNNs and ML models, the confusion matrices, and the accuracy for the validation and test datasets. A bagging ensemble method for each classifier was applied to the features generated on the penultimate layer [27]. The 5-fold cross-validation was established for optimally selecting the classifier.

Comparing the initial accuracy obtained on the test dataset, an improvement was obtained for SVM and RF, and the KNN remained the same. In this case, the performance was improved, and the two classifiers, SVM and RF, learned better from the features provided by our CNN. 

Both pre-trained EfficientNetV2B0 and DenseNet169 CNNs and the ML classifiers brought about improvements in the test dataset. SVM increased the accuracy for DenseNet169 from 0.891 to 0.986, and for EfficientNetV2B0, from 0.985 to 0.995. Furthermore, Table 6 shows that all classifiers exceeded the initial accuracy.

Other reliable metrics are the F1-score and MCC. The former assesses the predictive skill of a model’s performance, and the latter is more informative than F1-score and accuracy because it can be applied when classes are imbalanced. The processed dataset has 937 meningioma tumours and 500 healthy patients. For the same combination of EfficientNetV2B0 and SVM, the F1-score and MCC provide the highest values of 0.997 and 0.990, respectively. The results provided by the proposed CNN are comparable with pre-trained CNNs, and the difference is that the proposed CNN and RF work best, with an accuracy of 0.986 obtained on the test dataset.

**Figure 4:**
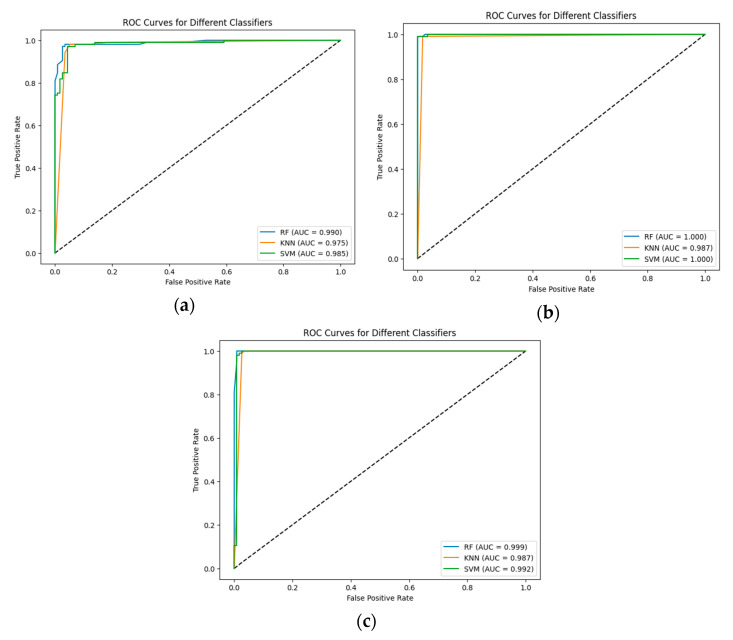
The ROC curves and area under the curve for each classifier: (**a**) Proposed CNN; (**b**) EfficientNetV2B0; and (**c**) DenseNet169.

The results were validated using the ROC curve. The ROC curves and area under the curve values for each CNN and classifier are shown in Figure 4.

The higher value of ROC-AUC refers to the better performance of the proposed hybrid models. In the present study, we observe that the pre-trained EfficientNetV2B0 has the best performance for initial accuracy in combination with all the proposed ML models, as confirmed by the ROC-AUC, which is 100% for RF and SVM. Also, the proposed CNN and RF are validated by an ROC-AUC of 0.99.

## 5. Comparison with the State-of-the-Art Models

The suggested hybrid models, which use three distinct classifiers—RF, SVM, and KNN—have demonstrated the highest results in accuracy and ROC-AUC on the brain MRI datasets, as previously discussed. It yielded promising findings, answering our original query about the utility of the proposed CNN model specifications and evaluating their usefulness by contrasting their classification performance with those of other pre-trained CNNs available in the literature. To achieve that, we need to evaluate their performance against a few ML classifiers that we discussed in our literature review. The procedures in the related work section were tested using the brain MRI dataset that is accessible on the Kaggle platform. We compared our suggested strategy in all three approaches (proposed CNN-ML, EfficientNetV2B0-ML, and DenseNet169-ML) with the results we obtained on the brain MRI dataset. Table 7 shows the results of our proposal and state-of-the-art models.

As can be seen, our proposals yield results that are similar to those shown in Table 5, and our approach produces the best accuracy when it comes to blends. DenseNet169-SVM (98.6%), CNN-RF (97.2%), and EfficientNetV2B0-SVM (99.5%) are the proposed models. The SVM classifier outperforms other classifiers in the suggested model and cutting-edge techniques, producing the greatest results when the SoftMax activation function is replaced with it. The EfficientNetV2B0-SVM combination outperforms the other pertinent experiments that employed ResNet50-SVM, AlexNet-SGD, and AlexNet-SSMO.

## 6. Conclusions

This study demonstrates the potential of hybrid CNN-ML models and a novel CNN-ML model for accurately classifying brain meningioma tumours from brain MRI images. ML classifiers, SVM, KNN, and RF, were efficiently used to classify features extracted from fully connected layers. Among the three models tested, the EfficientNetB0-SVM model achieved the highest classification accuracy of 99.5%. In addition, the proposed CNN model, together with the RF classifier, gives an accuracy of 97.2%. The proposed CNN-RF is an efficient model from the point of view of time and resources. Overall, we succeeded in accurately classifying meningioma brain tumours in this study by pointing out the importance of using a hybrid model.

Future work can be extended towards other types of images and pathologies and automated machine learning tools for feature classification in a tuning process. 

## Figures and Tables

**Table 1:** Comparative analysis with state-of-the-art works.

References	Dataset	Models	Performance Accuracy (%)	Limitations
[4], 2023	Brain MRI dataset, 3264 images.	Proposed CNNs ResNet-50, VGG16, InceptionV3	93.3 81.1 71.6 80	Alternative evaluation with ML tools is missed.
[5], 2023	Brain MRI dataset, 2213 images.	ResNet 50 and Inception V3	99.8	Only pre-trained CNNs are used.
[6], 2024	SARTAJ, and Br35H brain MRI dataset, 7023 images	Proposed CNNs	99	Pre-trained CNNs are not used.
[8], 2024	3264. Magnetic Resonance Imaging (MRI).	Proposed CNN–KNN	86	Pre-trained CNNs are not used.
[9], 2024	Brain MRI dataset, 7023 images.	Darknet19-SVM Darknet53-SVM Densenet201-SVM EfficientnetB0-SVM Resnet101-SVM Xception-SVM Proposed-KNN	95.58 96.87 97.81 97.93 95.87 96.23 97.15	A CNN with original architecture is missed.
[10], 2023	Brain MRI dataset, 7023 images.	AlexNet- BayesNet,(SMO), AlexNet–Naïve Bayes (NB)	88.75 98.15	Only a pre-trained CNN is proposed.
[11], 2023	Brain MRI dataset, 2950 images.	VGG-19	98.95	The study does not contain ML tools.
[12], 2024	Brain MRI dataset, 7023 images.	Proposed CNN–SVM	98	A study without pre-trained CNNs.
[13], 2021	Brain MRI dataset, 3064 images.	Proposed CNN	96	A study without pre-train CNNs and ML tools.
[14], 2023	Brain MRI dataset, 252 images.	DenseNet121	94.83	A study without ML tools.

**Table 2:** The parameters used for all CNNs.

CNNs	Total Parameters	Trainable Parameters	Non-Trainable Parameters
Proposed CNN	6.67 MB	0.67 MB	0
EfficientNetV2B0	23 MB	22.76 MB	236.75 KB
DenseNet169	48.77 MB	48.16 MB	618.75 KB
Proposed CNN	6.67 MB	0.67 MB	0

**Table 3:** Ablation process for proposed CNN.

Batches	Size Image	Epochs	Convolutional Layers
**20**	**80 × 80**	10	1
32	152 × 152	**15**	2
64	224 × 224	20	**3**

**Table 4:** Parameter values at each layer of the proposed CNN model.

Layer Name Activation	Activation Maps	Parameters
rescaling_1 (Rescaling)	(None, 80, 80, 3)	0
conv2d_3 (Conv2D)	(None, 80, 80, 32)	896
max_pooling2d_3 (MaxPooling2D)	(None, 40, 40, 32)	0
dropout_3 (Dropout)	(None, 40, 40, 32)	0
conv2d_4 (Conv2D)	(None, 40, 40, 64)	18,496
max_pooling2d_4 (MaxPooling2D)	(None, 20, 20, 64)	0
dropout_4 (Dropout)	(None, 20, 20, 64)	0
conv2d_5 (Conv2D)	(None, 20, 20, 128)	73,856
max_pooling2d_5 (MaxPooling2D)	(None, 10, 10, 128)	0
dropout_5 (Dropout)	(None, 10, 10, 128)	0
flatten_1 (Flatten)	(None, 12,800)	0
dense_2 (Dense)	(None, 128)	1,638,528
dense_3 (Dense	(None, 128)	16,512

**Table 5:** The established hyperparameters for MLs.

MLs	hyperparameters
RF	number of trees = 50 depth of trees = 100
KNN	n_neighbors = 4, class = KDTree
SVM	C = 0.1, gamma = 10

**Table 6:** Experimental results for combination CNNs-ML models.

Datasets	CNNs	MLs	ACC	F1-Score	MCC	Confusion Matrices [[TP FP] [ FN TN]]
Validation dataset	Proposed CNN	SVM	0.955	0.968	0.891	[[167 4] [ 7 65]]
		RF	0.950	0.965	0.880	[[167 4] [ 8 64]]
		KNN	0.946	0.962	0.871	[[166 5] [ 8 64]]
	DenseNet169	SVM	0.979	0.985	0.952	[[167 4] [ 1 71]]
		RF	0.9711	0.979	0.933	[[165 6] [ 1 71]]
		KNN	0.979	0.985	0.952	[[167 4] [ 1 71]]
	EfficientNetV2B0	SVM	0.995	0.996	0.991	[[115 0] [ 1 104]]
		RF	0.983	0.988	0.961	[[168 3] [ 1 71]]
		KNN	0.983	0.988	0.961	[[168 3] [ 1 71]]
Test dataset	Proposed CNN	SVM	0.950	0.951	0.902	[[106 9] [ 2 103]]
		**RF**	**0.972**	**0.974**	**0.946**	[[111 4] [ 2 103]]
		KNN	0.945	0.946	0.893	[[105 10] [ 2 103]]
	DenseNet169	**SVM**	**0.986**	**0.987**	**0.973**	[[112 3] [ 0 105]]
		RF	0.981	0.982	0.964	[[111 4] [ 0 105]]
		KNN	0.981	0.982	0.964	[[111 4] [ 0 105]]
	EfficientNetV2B0	**SVM**	**0.995**	**0.997**	**0.990**	[[171 0] [ 1 71]]
		RF	0.986	0.987	0.973	[[113 2] [ 1 104]]
		KNN	0.986	0.987	0.973	[[113 2] [ 1 104]]

**Table 7:** Comparison results with state-of-the-art CNN models.

State-of-the-Art Methods	CNNs-MLs	ACC
Siar at al. [34], 2019	Proposed CNN-DT	0.942
AlSaeed and Omar [35], 2022	ResNet50-SVM ResNet50-RF	0.92 0.857
Bohra and Gupta [36], 2022	VGG16-logistic regression classifier.	0.977
Sarkar et al. [10], 2023	AlexNet-BayesNet, AlexNet—Sequential Minimal Optimization (SMO) AlexNet—Naïve Bayes	0.887 0.981 0.862
Bansal et al. [12], 2023	Proposed CNN-SVM	0.99
Celik and Inik [9], 2024	Darknet19-SVM Darknet53-SVM Densenet201-SVM EfficientnetB0-SVM Resnet101-SVM Xception-SVM Proposed-KNN	0.955 0.968 0.978 0.979 0.958 0.962 0.971
Khushi et al. [37], 2024	AlexNet-Stochastic Gradient Descent (SGD)	0.987
Proposed hybrid CNN-ML	Proposed CNN-RF DenseNet169-SVM EfficientNetV2B0-SVM	0.972 0.986 0.995

## Data Availability

The data that support the findings of this study are openly available at https://data.mendeley.com/datasets/w4sw3s9f59/1 (accessed on 10 June 2024).
